# Individual cognitive change after DBS-surgery in Parkinson’s disease patients using Reliable Change Index Methodology

**DOI:** 10.1007/s40211-018-0271-4

**Published:** 2018-05-16

**Authors:** Thomas Foki, Daniela Hitzl, Walter Pirker, Klaus Novak, Gisela Pusswald, Johann Lehrner

**Affiliations:** 1 Department of Neurology, Medical University of Vienna, Währinger Gürtel 18-20, 1097 Vienna, Austria; 2 Department of Neurology, Wilhelminenspital Wien, Vienna, Austria; 3 Department of Neurosurgery, Medical University of Vienna, Vienna, Austria

**Keywords:** Parkinson, Deep brain stimulation, Cognition, Nucleus subthalamicus, Parkinson Krankheit, Tiefe Hirnstimulation, Kognition, Nucleus subthalamicus

## Abstract

Long-term therapy of Parkinson’s disease (PD) with levodopa (L-DOPA) is associated with a high risk of developing motor fluctuations and dyskinesia. Deep brain stimulation (DBS) in PD patients of the subthalamic nucleus can improve these motor complications. Although the positive effect on motor symptoms has been proven, postoperative cognitive decline has been documented. To tackle the impact of PD-DBS on cognition, 18 DBS patients were compared to 25 best medically treated Parkinson’s patients, 24 Mild Cognitive Impairment (MCI) patients and 12 healthy controls using the Neuropsychological Test Battery Vienna-long (NTBV-long) for cognitive outcome 12 months after first examination. Reliable change index methodology was used. Overall, there was cognitive change in individual patients, but the change was very heterogeneous with gains and losses. Further research is needed to identify the mechanisms that lead to improvement or deterioration of cognitive functions in individual cases.

## Zusammenfassung

Die Langzeittherapie der Parkinson-Krankheit mit Levodopa (L-DOPA) ist mit einem hohen Risiko für die Entwicklung motorischer Fluktuationen und Dyskinesien verbunden. Die tiefe Hirnstimulation des Nucleus subthalamicus bei Parkinson-Patienten (PD-DBS) kann diese motorischen Komplikationen verbessern. Obwohl der positive Effekt auf die motorischen Symptome nachgewiesen wurde, wurden postoperative kognitive Verschlechterungen dokumentiert. Um den Einfluss von PD-DBS auf die Kognition zu untersuchen, wurden 18 DBS-PD-Patienten mit 25 am besten medizinisch behandelten PD-Patienten, 24 Patienten mit leichter kognitiver Beeinträchtigung (MCI) und 12 gesunden Kontrollpersonen 12 Monate nach der ersten Untersuchung mittels der neuropsychologischen Testbatterie Vienna-long (NTBV-long) verglichen. Dabei wurde die Reliable Change-Index-Methodik verwendet. Insgesamt gab es kognitive Veränderungen bei einzelnen Patienten, aber die Veränderung war sehr heterogen, mit Gewinnen und Verlusten. Weitere Untersuchungen sind erforderlich, um die Mechanismen zu identifizieren, die in einzelnen Fällen zur Verbesserung oder Verschlechterung kognitiver Funktionen postoperativ führen.

## Introduction

Deep brain stimulation (DBS) is an established therapy for advanced Parkinson’s disease (PD). The procedure alleviates tremor, rigidity, bradykinesia and levodopa-induced dyskinesia. The effectiveness of bilateral DBS of the subthalamic nucleus (STN) on motor symptoms in patients with advanced PD is accepted. While STN-DBS can significantly improve motor symptoms in PD patients, adverse cognitive effects have also been reported [1]. The specific effects of STN-DBS on cognitive function and the related mechanisms remain unclear.

An early meta-analysis dealing with the cognitive squeal of STN-DBS in PD revealed significant, albeit small, declines in executive functions, verbal learning and memory. Moderate declines were only reported in semantic and phonemic verbal fluency [2]. A more recent meta-analysis concluded that a progressive decrease in verbal fluency after STN-DBS is consistently reported and, although executive function is unchanged in the intermediate stage postoperatively, it tends to decline in the early and later stages [3]. Another recent meta-analysis investigating cognition after deep brain stimulation in PD found small declines in psychomotor speed, memory attention, executive functions and overall cognition and moderate declines were found in both semantic and phonemic fluency [4]. A recent focused review confirmed the worsening of verbal fluency after DBS [5]. The most recent meta-analysis suggested that STN-DBS results in decreased global cognition, memory, verbal fluency and executive function compared to a PD control group. No significant difference was found in other cognitive domains. STN-DBS seems relatively safe with respect to cognitive function, and future studies should focus on the exact mechanisms of possible verbal deterioration after surgery [6].

Every meta-analysis included studies that were repeated measure designs that used neuropsychological tests as dependent variables, many of which are susceptible to measurement artefacts. The reports discussed the problem of the influence of practice effects and regression to the mean effects in repeated testing designs and suggested that the impact of practice effects should be assessed and future studies could be enhanced by assessing un-operated best medically treated (BMT) patients, clinical control groups and normal controls at similar time intervals and statistically controlling for typical practice effects [2, 4].

The gold standard to evaluate cognitive outcome are randomized controlled studies. However, randomized controlled study methodology is not suitable for assessing cognitive outcome in single patients in the clinical setting. In order to predict cognitive outcome in single patients, the reliable change index (RCI) methodology has been developed [7]. RCI is used to examine the influence of disease progression over time and assesses performance across time accounting for changes due to factors not related to cognitive impairments. Changes of outcome results due to practice effects have to be differentiated from changes due to disease effects or intervention effects. In serial testing procedures improvement in test performance can be achieved solely through practice effects. On the other hand, effects of disease progression and aging may influence test outcome. Furthermore, effects of therapeutic interventions must be considered. Because of the neurodegenerative nature of PD, progressive changes in neuropsychological testing in individual cases over time have to be expected.

Recent studies have investigated the effect of DBS on cognition using RCI methodology. In general, significant cognitive deterioration was found only in a few PD-DBS patients suggesting that neuropsychological evaluations may identify possible mild cognitive changes following surgery [8–15].

As discussed above, an open question is the influence of practice effects and regression to the mean effects in repeated testing designs in DBS surgery of PD patients. Using un-operated medically best treated PD controls, clinical control groups and normal controls with similar test-retest intervals offers the opportunity to investigate practice effects of DBS surgery on cognitive changes above that of PD progression and clinical intervention effects. In a recent study our group showed by using the Neuropsychological Test Battery Vienna short version (NTBV-short) that roughly 10% of DBS patients showed cognitive decline 12 months after the first examination, mainly affecting the domains attention and executive functioning (phonemic fluency) using reliable change index methodology [16]. However, in our prior study only cognitive domains and not specific tests were used and as the NTBV-short uses only a limited number of neuropsychological tests a more extensive neuropsychological test battery covering a greater range of functions would be desirable. Therefore, the goal of the present study was to investigate cognitive change in single patients after DBS in PD in relation to PD-BMT controls, MCI patients and healthy controls using single variables of the NTBV long and reliable change index methodology.

## Patients and methods

The current data are part of a larger research project, the Vienna Mild Cognitive Impairment and Cognitive Decline in Parkinson’s Disease Study (VMCI-CD-PD Study). The VMCI-CD-PD Study is a prospective cohort study including consecutive, community-dwelling PD patients who attend the movement disorder clinic for assessment of their Parkinsonism. The study protocol was in accordance with the Helsinki Declaration and approved by the Ethical Committee of the Medical University of Vienna.

All PD patients underwent clinical examination and neuropsychological testing. The clinical assessment encompassed a complete medical history, a detailed history of PD, which was obtained using a standardized interview, and a complete neurological examination including the motor section of the Unified Parkinson’s Disease Rating Scale (UPDRS-III) [17] and the modified Hoehn and Yahr scale [18]. Clinical examination and neuropsychological testing were performed during the “on-state”. Neuropsychological baseline testing was performed before DBS surgery, and as a consequence, before starting stimulation. Patients who had never undergone computed tomography (CT) or magnetic resonance imaging (MRI) during the course of PD and patients showing clinical features incompatible with previous imaging results were referred to structural imaging. Both neuroimaging and clinical features were used to determine significant cerebrovascular disease or other co-morbid conditions with a potential impact on cognitive outcomes.

Inclusion and exclusion criteria were similar to those used in other studies. All PD patients had to fulfill UK Parkinson’s Disease Society Brain Bank criteria [19] for probable PD. Inclusion and exclusion criteria were specified as follows. PD-DBS patients were suffering from PD for at least 5 years with a positive response to L‑DOPA or Apomorphin treatment. All PD-DBS patients showed not manageable motor symptoms like dyskinesia, tremor or fluctuations. Excluded were patients with a secondary Parkinson syndrome or other degenerative processes with Parkinson like symptoms. Severe cognitive impairments like dementia or psychiatric disorders were exclusion criteria. Psychiatric diagnoses were based on psychiatric diagnostic interviews performed by a psychiatrist. Exclusion criteria were not re-examined before second testing. Any comorbidities and structural brain lesions that would interfere with the surgical procedure were ruled out. Controls and patients were excluded from the study if any of the following conditions applied: (a) evidence of having had a stroke as determined by neuroradiologic and clinical examination, (b) history of severe head injury, (c) current psychiatric diagnosis according to ICD-10 with the exception of patients with (sub)depressive symptoms, (d) any medical condition that can lead to cognitive deterioration including renal, respiratory, cardiac and hepatic disease, or (e) a diagnosis of dementia according to DSM IV [20]. Patients were assessed on their regular medication and were required to have a Mini-Mental State Examination (MMSE) [21] score of ≥26. There were no study withdrawals and participants were enrolled consecutively.

For the assessment of neurocognitive functioning all participants were subjected the long version of the Neuropsychological Test Battery Vienna (NTBV) (meduniwien.ac.at/kpfg). The NTBV assesses several cognitive domains including attention, executive functioning, language and memory domains with corresponding z‑scores for single neuropsychological measurers [22]. A total z‑score across all measurers is also available [22]. The Alters-Konzentrations-Test (AKT) [23], the number-symbol-test, the Trail Making Test B (TMT-B) [24, 25] and the symbol counting task from the cerebral insufficiency test (C. I.) [26] were used to assess attention. Executive functions were investigated using the Trail Making Test A (TMT-A) [25], the Maze Test from the NAI Test Battery [27], the interference test from the C. I. [26], the color-word-test from the NAI Test Battery [27] and the five-point-test [28]. Naming as many words as possible beginning with the letters f, b and l within one minute for each task was used to tap lexical verbal fluency [29]. In order to test language functions, a verbal fluency task, naming as many animals, groceries and tools as possible within one minute per task [29], and a confrontation naming task, the Boston Naming Test [30], were used. Episodic memory was tested using the Verbal Selective Reminding Test (VSRT) with the subtests of immediate recall, total recall, delayed recall and recognition [31]. After the completion of the evaluation, the cognitive status was determined according to age and education corrected norms using a normative sample of cognitively healthy controls. For this purpose, the flexible GAMLSS (Generalized Additive Models for Location, Scale and Shape) model class was used [22]. The NTBV was performed two times. The second testing was performed one year after the first.

The study included seventy-seven participants subdivided into four groups. Patients with PD were divided into a PD-DBS group and a PD-BMT group (best medical treatment only). The DBS group underwent deep brain stimulation after the first testing. Patients received bilateral MR-based, stereotactic DBS surgery. The optimum electrode position within the STN was assessed intraoperative at the awake patient, applying macrostimulation to test for motor improvement and side effects. In addition, postoperative MR imaging was used to exclude peri-operative structural abnormalities and to reassure the correct electrode positions. Starting with standard DBS parameters (60 microseconds impulse duration at 130 Hz), the voltage was gradually increased and stimulation parameters individually adjusted. In parallel, dopaminergic therapy was gradually decreased, as far as tolerated by the patient [32, 33].

Community-dwelling patients complaining of cognitive problems who came to the memory outpatient clinic for assessment of a possible cognitive disorder were included in the study. MCI was defined according to the Petersen criteria [34].

The healthy control group consisted of participants without PD and cognitive impairments. Great care was taken enrolling a sufficient number of cognitively healthy control subjects living independently at home. Control subjects were recruited by means of advertisements. They underwent a screening evaluation using a standardized clinical interview and cognitive screening. Imaging procedures, neurological examination, standard laboratory blood tests and informant reports were not included in the evaluation. They were assessed as being in good health. Criteria for healthy function were identified as being similar to those in the Mayo research studies [35]: (a) no active neurological or psychiatric disease, (b) no psychotropic medications, and (c) the subjects may have medical disorders but neither they nor their treatment compromises cognitive function. Cognitive status was given special attention and cognitively healthy control subjects were screened for intact cognition. They were required to have an MMSE [21] score greater than or equal to 27 and a MOCA [36] score greater than or equal to 26 adjusted for education. Control subjects did not overtly complain about cognitive problems.

All four patient groups were similar in terms of age, gender, Mini-Mental State Examination (MMSE) status [21], verbal intelligence (WST) [37] and depressive symptoms (BDI-II) [38]. Using Kruskal-Wallis analyses no statistical group differences were found (all *p*’s > 0.3). Demographic and clinical data are shown in Table 1.

**Table 1:** Demographic and clinical data across different groups

	Total group (*N* = 77)	Healthy control (*N* = 12)	MCI (*N* = 24)	PD-BMT (*N* = 25)	PD-DBS (*N* = 16)
Age	62.9 ± 7.7	65.1 ± 5.7	63.1 ± 7.4	62.9 ± 6.5	59.3 ± 10.6
Education	10.1 ± 2.3	9.9 ± 2.3	10.0 ± 2.3	10.4 ± 2.35	10.1 ± 2.4
Sex (m/w)	37/42	3/9	14/10	14/11	5/11
MMSE	28.3 ± 1.3	28.8 ± 0.8	28.1 ± 1.2	28.4 ± 1.2	28.0 ± 1.7
WST-IQ	103.9 ± 10.0	103.0 ± 7.5	104.5 ± 9.9	103.2 ± 10.6	105.6 ± 12.4
BDI-II	10.2 ± 6.0	10.7 ± 5.8	10.3 ± 5.4	9.3 ± 6.1	11.0 ± 6.8
UPDRS motor score	–	–	–	28.4 ± 12.7	25.4 ± 11.4
Hoehn & Yahr scale	–	–	–	3.68 ± 1.9	3.7 ± 2.0

*MMSE* Mini Mental State Examination, *WST-IQ* Wortschatztest, *BDI-II* Beck Depressions Inventar, *UPDRS* The Unified Parkinson’s Disease Rating Scale

## Statistical Analyses

Based on the test z‑scores for each cognitive measure, standard deviations were calculated for each cognitive measure separated by group [7]. Mean test z‑scores and standard deviations are used for calculating the Reliable Change Index (RCI) as follows:

$$RCI=((X_{2}-X_{1})-(M_{2}-M_{1}))\times SED$$

The difference of (X2 − X1) characterizes the individual test scores of a participant at the two test sessions over time. M2 and M1 represent the mean scores of the group the patient is compared to. The calculation of the critical difference (SED) uses the standard deviation and test-retest reliability of the specific comparison group. The standard deviation is taken from the first test session. The retest-reliability coefficient of a given test is calculated by using test scores of the first test session (X1) and test scores of the second test session (X2).

$$SED=\sqrt{2\times \mathrm{SEM}^{2}}$$

$$SEM=SD\sqrt{(1-r_{tt}})$$

This formula is based on [7] and a significant change can be assumed at a change of ±1.64 (*p* = 0.05). Based on this, the confidence interval is calculated to determine a minimum and a maximum limit that represent significant change.

In order to ensure replicability for future studies the calculations are shown using a case example. A patient taken from the PD-DBS group is compared to the PD-BMT group for verbal memory delayed recall. All scores have already been transformed into z‑scores. The calculation process is divided into two steps. At first, calculation of the confidence interval using mean score and standard deviation of the PD-BMT group are calculated for both test sessions. Mean score of the verbal memory delayed recall at first testing session was 0.05 with a standard deviation of 0.82 for the PD-BMT group. Mean score of the verbal memory delayed recall at second testing session was −0.36 with a standard deviation of 1.35 for the PD-BMT group. The test-retest reliability coefficient (rtt = 0.71) results from the correlation of the test scores across time for the PD-BMT group. The formula for the standard error of measurement (SEM) is derived by including the test-retest correlation coefficient and standard deviation from the PD-BMT group.

$$SEM=0.82 \times\sqrt{\left(1-0.71\right)}=0.44$$

Based on the standard error of measurement the standard error of difference (SED) is calculated.

$$SED=\sqrt{\left(2*0.44^{2}\right)=0.62}$$

According to [7] a deviation of ±1.64 can be assumed as statistically significant.

$$\text{CI}\, =1.64*0.62=1.02$$

The upper and lower scores of the confidence interval are serving as limiting values for defining statistical significance. After subtracting the second and the first mean score (M2 − M1) the confidence interval is added or subtracted. Difference of mean scores (DM): M2 − M1 = −0.36 − 0.05 = −0.41; Limiting value deterioration: DM − CI = −0.41 − 1.02 = −1.43; Limiting value improvement: DM + CI = −0.41 + 1.02 = 0.61. A patient’s RCI has to be higher or lower than the limiting values in order to detect a statistically significant change between the first and second testing. The scores of reliability coefficient, standard error of measurement and standard error of a difference are calculated like shown above. Thus, for the verbal memory delayed recall the figures are as follows: rtt = 0.7; SEM = 0.44; SED = 0.62; M1 = 0.05; M2 = −0.36. Limiting value deterioration: −1.43; Limiting value improvement: 0.61.

The patient chosen for the illustrative example reached a z-score of −0.9 (X1) in the first and −1.2 (X2) in the second testing of verbal memory delayed recall. The mean scores of the group and the individual test scores are put in the formula for calculating the reliable change index: RCI = (((−1.2) − (−0.9)) − ((−0.482) − (−0.075))) * 0.62 = 0.066. This results in a RCI score of 0.07 for this patient. This value is compared with the limiting values calculated above. The limiting values of 0.61 and −1.43 are not surpassed or undercut. Therefore, no significant change of memory ability in this patient in comparison to the Parkinson BMT group has been detected.

## Results

In order to determine the individual performance using RCI methodology, RCI calculations were performed for each participant in each group for every cognitive measure. The scores of the reliability coefficient, standard error of measurement, standard error of difference and the limiting values for improvement and deterioration as calculated for the PD-BMT group, healthy control group and MCI group are shown in Table 2, 3 and 4, respectively. Table 5 shows the results for the RCI analysis comparing the PD-DBS group to the calculated limiting values of the PD-BMT group, the control group and the MCI group, respectively.

**Table 2:** rtt, SEM, SED and limiting values for the PD-BMT group

Test	Mean score and SD 1st testing	Mean score and SD 2nd testing	rtt	SEM	SED	Limiting value deterioration	Limiting value improvement
AKT time	−0.78 ± 1.74	−1.10 ± 1.47	0.80	0.79	1.11	−2.14	1.51
AKT total/time	−0.72 ± 1.69	−1.03 ± 1.39	0.82	0.72	1.02	−1.99	1.36
Digit-Symbol -Test (WAIS-R)	−0.48 ± 1.06	−0.86 ± 0.83	0.75	0.53	0.75	−1.61	0.86
Symbols counting (C. I.)	−0.39 ± 1.23	−0.83 ± 1.17	0.55	0.83	1.17	−2.36	1.48
Trail Making Test – TMTB	−0.63 ± 1.42	−1.10 ± 1.68	0.74	0.73	1.03	−2.15	1.22
TMTB – TMTA difference	−0.30 ± 1.31	−0.72 ± 1.31	0.82	0.79	1.11	−2.25	1.39
Semantic verbal fluency SWT total words	−0.02 ± 1.28	−0.32 ± 1.46	0.79	0.59	0.83	−1.67	1.06
Boston Naming Test (mBNT)	−0.58 ± 0.57	−0.53 ± 0.63	0.45	0.42	0.60	−0.92	1.04
Phonematic Verbal Fluency PWT total words	−0.48 ± 1.47	−0.58 ± 1.68	0.75	0.73	1.03	−1.80	1.60
Verbal memory immediate recall (VSRT)	−0.16 ± 0.69	−0.66 ± 0.82	0.36	0.55	0.78	−1.78	0.78
Verbal memory total recall (VSRT)	0.07 ± 0.77	−0.28 ± 0.81	0.58	0.50	0.71	−1.51	0.81
Verbal memory delayed recall (VSRT)	0.05 ± 0.82	−0.36 ± 1.35	0.71	0.44	0.62	−1.43	0.62
Verbal memory recognition (VSRT)	−0.71 ± 0.64	−0.54 ± 0.70	0.06	0.62	0.88	−1.27	1.60
Stroop color words – colors	−0.48 ± 1.32	−0.52 ± 1.17	0.25	1.14	1.62	−2.70	2.62
Stroop color words-words	−0.39 ± 1.09	−0.33 ± 1.10	0.42	0.83	1.18	−1.87	1.99
Stroop total/time	−0.39 ± 1.03	−0.48 ± 0.98	0.34	0.84	1.18	−2.03	1.85
Stroop color words-difference words colors	0.30 ± 1.01	0.24 ± 1.28	0.51	0.71	1.00	−1.70	1.58
Interference (C. I.) time	−0.57 ± 1.23	−0.78 ± 1.31	0.80	0.55	0.77	−1.48	1.06
Interference (C. I.) total/time	−0.56 ± 1.13	−0.67 ± 1.17	0.75	0.56	0.79	−1.40	1.19
Nonverbal Fluency Five Point Test – total correct	−0.35 ± 1.09	−0.04 ± 0.80	0.67	0.63	0.89	−1.15	1.77
Nonverbal Fluency Five Point Test – perseverations	−0.30 ± 0.90	−0.18 ± 0.91	0.45	0.67	0.94	−1.43	1.67
Planning Maze Test – NAI time	−0.82 ± 1.57	−0.58 ± 1.25	0.44	1.17	1.66	−2.48	2.95
Planning Maze Test – NAI total/time	−0.58 ± 1.20	−0.43 ± 1.06	0.54	0.82	1.15	−1.75	2.04
Trail Making Test – TMTA	−0.68 ± 1.28	−0.65 ± 1.16	0.66	0.75	1.06	−1.71	1.76
NTBV Total Score	−0.36 ± 0.64	−0.49 ± 0.62	0.89	0.21	0.30	−0.62	0.36

*rtt* test-retest reliability, *SEM* standard error of measurement, *SED* standard error of difference, *PD-BMT* Parkinson’s Disease best medically treated;

**Table 3:** rtt, SEM, SED and limiting values for the MCI group

Test	Mean score and SD 1st testing	Mean score and SD 2nd testing	rtt	SEM	SED	Limiting value deterioration	Limiting value improvement
AKT time	−0.29 ± 1.52	−0.19 ± 1.33	0.69	0.85	1.21	−1.88	2.07
AKT total/time	−0.29 ± 1.48	−0.22 ± 1.31	0.66	0.86	1.22	−1.92	2.06
Digit-Symbol-Test (WAIS-R)	−0.04 ± 1.06	−0.16 ± 1.04	0.81	0.46	0.65	−1.19	0.96
Symbols counting (C. I.)	−0.02 ± 1.49	0.07 ± 1.49	0.74	0.76	1.07	−1.67	1.84
Trail Making Test - TMTB	−0.25 ± 1.41	−0.96 ± 1.70	0.76	0.68	0.97	−2.29	0.88
TMTB – TMTA difference	−0.09 ± 1.29	−0.64 ± 1.37	0.71	0.69	0.98	−2.16	1.05
Semantic verbal fluency SWT total words	−0.07 ± 1.15	−0.33 ± 1.58	0.59	0.74	1.04	−1.97	1.46
Boston Naming Test (mBNT)	−1.11 ± 0.92	−0.99 ± 0.91	0.73	0.48	0.68	−0.99	1.22
Phonematic Verbal Fluency PWT total words	−0.82 ± 1.49	−0.75 ± 1.47	0.60	0.94	1.33	−2.12	2.26
Verbal memory immediate recall (VSRT)	−0.24 ± 1.14	−0.83 ± 0.96	0.53	0.78	1.10	−2.39	1.21
Verbal memory total recall (VSRT)	−0.33 ± 0.97	−0.72 ± 1.07	0.61	0.60	0.85	−1.79	1.01
Verbal memory delayed recall (VSRT)	−0.51 ± 0.97	−1.16 ± 1.62	0.71	0.53	0.74	−1.87	0.57
Verbal memory recognition (VSRT)	−0.87 ± 1.13	−1.32 ± 1.36	0.55	0.76	1.07	−2.20	1.30
Stroop color words - colors	0.15 ± 1.12	−0.04 ± 1.42	0.52	0.78	1.10	−1.99	1.61
Stroop color words-words	−0.09 ± 1.06	−0.27 ± 1.22	0.74	0.54	0.76	−1.42	1.07
Stroop total/time	−0.11 ± 1.07	−0.21 ± 1.16	0.74	0.54	0.77	−1.36	1.15
Stroop color words-difference words colors	0.11 ± 1.21	0.30 ± 1.11	0.68	0.69	0.97	−1.40	1.78
Interference (C. I.) time	−0.01 ± 1.34	−0.05 ± 1.61	0.70	0.73	1.04	−1.74	1.66
Interference (C. I.) total/time	0.00 ± 1.30	0.05 ± 1.35	0.77	0.63	0.89	−1.41	1.51
Nonverbal Fluency Five Point Test - total correct	−0.29 ± 1.42	0.28 ± 1.22	0.55	0.95	1.34	−1.63	2.76
Nonverbal Fluency Five Point Test - perseverations	−0.37 ± 0.87	−0.23 ± 1.00	0.01	0.86	1.22	−1.86	2.14
Planning Maze Test – NAI time	0.33 ± 1.47	−0.04 ± 1.44	0.78	0.69	0.97	−1.97	1.21
Planning Maze Test – NAI total/time	0.35 ± 1.20	−0.04 ± 1.18	0.75	0.60	0.84	−1.78	0.99
Trail Making Test - TMTA	−0.26 ± 1.16	−0.27 ± 1.34	0.36	0.92	1.30	−2.15	2.13
NTBV Total Score	−0.27 ± 0.58	−0.43 ± 0.74	0.69	0.32	0.46	−0.90	0.60

*rtt* test-retest reliability, *SEM* standard error of measurement, *SED* standard error of difference, *MCI* Mild cognitive impairment

**Table 4:** rtt, SEM, SED and limiting values for the healthy control group

Test	Mean score and SD 1st testing	Mean score and SD 2nd testing	rtt	SEM	SED	Limiting value deterioration	Limiting value improvement
AKT time	0.76 ± 1.42	0.24 ± 0.90	0.24	1.24	1.75	−3.39	2.34
AKT total/time	0.76 ± 1.37	0.27 ± 0.90	0.24	1.19	1.68	−3.25	2.26
Digit-Symbol -Test (WAIS-R)	0.28 ± 0.97	0.20 ± 0.97	0.82	0.42	0.59	−1.04	0.89
Symbols counting (C. I.)	0.67 ± 0.86	0.20 ± 1.20	0.32	0.71	1.01	−2.12	1.18
Trail Making Test - TMTB	−0.21 ± 1.10	0.07 ± 1.06	0.63	0.67	0.94	−1.27	1.83
TMTB – TMTA difference	−0.15 ± 0.99	0.04 ± 1.05	0.27	0.85	1.20	−1.78	2.15
Semantic verbal fluency SWT total words	0.64 ± 1.16	0.52 ± 1.02	0.85	0.45	0.64	−1.17	0.93
Boston Naming Test (mBNT)	−0.71 ± 0.69	−0.55 ± 0.54	0.59	0.44	0.62	−0.87	1.18
Phonematic Verbal Fluency PWT total words	0.21 ± 0.87	0.35 ± 0.86	0.76	0.43	0.61	−0.86	1.13
Verbal memory immediate recall (VSRT)	0.02 ± 1.10	−0.35 ± 0.79	0.17	1.01	1.42	−2.70	1.97
Verbal memory total recall (VSRT)	0.48 ± 0.82	0.01 ± 0.63	0.39	0.64	0.90	−1.96	1.00
Verbal memory delayed recall (VSRT)	0.22 ± 0.92	−0.37 ± 1.07	0.11	0.86	1.22	−2.58	1.42
Verbal memory recognition (VSRT)	−0.81 ± 0.63	−0.71 ± 1.07	0.19	0.56	0.80	−1.21	1.41
Stroop color words - colors	0.83 ± 0.93	0.41 ± 1.13	0.54	0.63	0.89	−1.88	1.03
Stroop color words-words	0.13 ± 0.85	0.05 ± 0.21	0.71	0.46	0.65	−1.14	0.99
Stroop total/time	0.05 ± 0.77	−0.12 ± 0.77	0.65	0.46	0.65	−1.23	0.89
Stroop color words-difference words colors	0.33 ± 0.71	0.21 ± 0.55	0.61	0.44	0.63	−1.15	0.90
Interference (C. I.) time	0.28 ± 0.95	0.58 ± 1.09	0.50	0.67	0.95	−1.27	1.85
Interference (C. I.) total/time	0.35 ± 1.03	0.67 ± 1.12	0.49	0.73	1.03	−1.37	2.02
Nonverbal Fluency Five Point Test - total correct	0.47 ± 0.93	0.30 ± 1.10	0.48	0.67	0.95	−1.73	1.39
Nonverbal Fluency Five Point Test - perseverations	−0.60 ± 1.01	−0.34 ± 0.82	0.58	0.66	0.93	−1.27	1.79
Planning Maze Test – NAI time	0.92 ± 1.09	1.15 ± 0.92	0.43	0.82	1.16	−1.66	2.14
Planning Maze Test – NAI total/time	0.93 ± 0.91	0.95 ± 0.86	0.38	0.71	1.01	−1.63	1.68
Trail Making Test - TMTA	−0.11 ± 1.22	0.25 ± 0.97	0.64	0.73	1.04	−1.34	2.06
NTBV Total Score	0.24 ± 0.46	0.18 ± 0.43	0.72	0.24	0.34	−0.63	0.50

*rtt* test-retest reliability, *SEM* standard error of measurement, *SED* standard error of difference

**Table 5:** Results of individual comparison between PD-DBS group and PD-BMT group, MCI group and healthy control group

Test	Mean score and SD 1st testing PD−DBS	Mean score and SD 2nd testing PD−DBS		PD-BMT group			MCI group			Control group	
			% Deteriorated	% No change	% Improved	% Deteriorated	% No change	% Improved	% Deteriorated	% No change	% Improved
AKT time	−0.89 ± 1.42	−2.08 ± 1.75	6.2	81.3	12.5	6.2	87.5	6.2	6.2	81.2	12.5
AKT total/time	−0.84 ± 1.39	−1.96 ± 1.64	6.2	81.3	12.5	6.2	87.5	6.2	6.2	81.2	12.5
Digit-Symbol -Test (WAIS-R)	−1.22 ± 0.75	−1.28 ± 0.70	6.2	93.7	0	0	100	0	0	100	0
Symbols counting (C. I.)	−0.89 ± 1.06	−0.86 ± 1.33	0	81.2	12.5	12.5	81.2	6.2	6.2	81.2	12.5
Trail Making Test - TMTB	−1.83 ± 1.57	−2.08 ± 1.58	6.2	68.8	25	0	69	31	6.2	93.7	0
TMTB – TMTA difference	−1.27 ± 1.13	−1.58 ± 0.96	12.5	68.8	18.7	0	75.2	24.8	6.2	87.5	6.2
Semantic verbal fluency SWT total words	0.01 ± 1.14	−0.85 ± 0.94	6.2	81.2	12.5	12.5	75.5	12.5	0	100	0
Boston Naming Test (mBNT)	−0.96 ± 0.87	−0.74 ± 0.69	6.2	93.7	0	0	100	0	0	100	0
Phonematic Verbal Fluency PWT total words	−0.48 ± 1.20	−1.12 ± 1.44	6.2	75.1	18.7	6.2	75.1	18.7	0	100	0
Verbal memory immediate recall (VSRT)	−0.50 ± 0.97	−0.69 ± 1.26	0	75	25	0	87.5	12.5	6.2	81.2	12.5
Verbal memory total recall (VSRT)	−0.14 ± 1.17	−0.64 ± 1.17	0	93.7	6.2	6.2	81.2	12.5	0	100	0
Verbal memory delayed recall (VSRT)	−0.08 ± 1.44	−0.48 ± 1.48	0	81.2	18.7	6.2	56.6	37.2	6.2	75	18.7
Verbal memory recognition (VSRT)	−1.11 ± 0.79	−0.76 ± 0.66	6.2	81.2	12.5	6.2	75.1	18.7	0	100	0
Stroop color words - colors	−0.58 ± 0.98	−0.98 ± 1.41	12.5	75.5	12.5	12.5	81.2	6.2	6.2	93.7	0
Stroop color words-words	−1.03 ± 0.90	−1.15 ± 1.37	0	93.7	6.2	6.2	87.5	6.2	0	100	0
Stroop total/time	−0.90 ± 0.84	−0.95 ± 1.31	0	93.7	6.2	0	93.7	6.2	0	100	0
Stroop color words-diference words colors	0.78 ± 1.19	0.91 ± 1.42	6.2	93.7	0	18.7	81.2	0	0	100	0
Interference (C. I.) time	−0.55 ± 1.17	−1.01 ± 1.26	6.2	81.2	12.5	6.2	87.5	6.2	6.2	93.7	0
Interference (C. I.) total/time	−0.49 ± 1.12	−0.89 ± 1.15	0	87.5	12.5	6.2	87.5	6.2	6.2	93.7	0
Nonverbal Fluency Five Point Test - total correct	−0.96 ± 1.97	−0.56 ± 1.11	0	93.7	6.2	18.7	68.8	12.5	6.2	87.5	6.2
Nonverbal Fluency Five Point Test - perseverations	−0.19 ± 0.94	−0.28 ± 0.74	12.5	87.5	0	12.5	75	12.5	0	100	0
Planning Maze Test – NAI time	−0.36 ± 1.28	−0.95 ± 1.28	31	38	31	6.2	68.8	25	6.2	87.5	6.2
Planning Maze Test – NAI total/time	−0.28 ± 0.93	−0.79 ± 1.11	12.5	81.3	6.2	0	87.5	12.5	6.2	93.7	0
Trail Making Test - TMTA	−1.04 ± 0.79	−0.72 ± 0.80	6.2	81.3	12.5	6.2	81.3	12.5	12.5	87.5	0
NTBV Total Score	−0.54 ± 0.62	−0.86 ± 0.62	0	100	0	0	100	0	0	100	0

The comparison of the PD-DBS group to the PD-BMT group indicated deterioration for the cognitive measure of Planning Maze Test—NAI time with 31% of patients showing a significant deterioration. However, improvements have also been found such as that 31% of patients had improved Planning Maze Test—NAI time performance, 18.7% of patients had improved in TMTB – TMTA difference score; 18.7% of patients improved in Phonematic Verbal Fluency PWT total words and 18.7% of patients improved in verbal memory delayed recall. No changes were found for the NTBV total score. The results of the comparison are displayed in Table 5.

When comparing PD-DBS patients to MCI patients a similar heterogeneous picture emerged. For Stroop color words-difference (18.7%), Nonverbal Fluency Five Point Test—total correct (18.7%) significant deteriorations have been detected. On the other side, a larger number of patients improved with 24.8% in TMTB – TMTA difference score, 37.2% in verbal memory delayed recall, 25.0% in Planning Maze Test – NAI time. No changes were found for the NTBV total score. The results of the comparison are displayed in Table 5.

The comparison of the PD-DBS group with the healthy control group revealed similar heterogeneous results compared to the other groups. For the Trail Making Test – TMTA (12.5%) significant deteriorations have been detected. As with the other comparisons, 18.7% of patients improved in verbal memory delayed recall, with little improvements in other tests. No changes were found for the NTBV total score. The results of the comparison are displayed in Table 5.

## Discussion

In order to investigate cognitive changes in patients with PD with DBS we compared the neuropsychological profile of four groups, PD patients with DBS, PD patients best medically treated, MCI patients and healthy controls at two time points with re-examination twelve months later. In terms of comparing the PD-DBS group to the three remaining groups at the two test sessions a reliable change index was calculated for every cognitive measure.

Whereas no significant changes for the NTBV total score were found, there was cognitive change in specific tests in individual patients, but the change was very heterogeneous with gains and losses. When comparing the two Parkinson groups it was interesting to find a decline regarding specific executive functions, such as planning, in single patients in the PD-DBS group. However, in a third of the PD-DBS patients improvements have been found in planning capability and a fifth of PD-DBS patients phonematic verbal fluency and verbal memory delayed recall improved. In all other cognitive measures most patients experienced no change. The finding of a relatively large degree of individual variation corroborates earlier reports using RCI methodology in PD-DBS patients [8–15].

The most recent meta-analysis suggested that STN-DBS results in decreased memory, verbal fluency, and executive function compared to a PD control group. No significant differences were found in other cognitive domains, and thus supporting our results regarding cognitive deterioration [6]. However, according to our results, many PD patients do also improve after STN-DBS. The reason why some patients show decline and others show gains in cognition after STN-DBS is not clear and should be the target of future research. One could think of effects of preoperative clinical, cognitive or affective status, pre- and postoperative medication, postoperative stimulation procedure, postoperative differences in motivation for neurocognitive testing and different anatomical location of the electrodes. Based on our results and the results of other RCI studies there appears to be heterogeneity in the prevalence of worsening and gaining effects after DBS in PD. What is consistently reported as a group-effect seems to be mainly driven by a small, but substantial, subgroup of DBS-treated patients [5].

One important goal of the present study was to compare neurocognitive outcome of PD-DBS with other groups than PD patients using single tests because up to date no such study has been performed yet. The only study providing such a comparison used neurocognitive domains but no single tests [16]. Such an investigation is important in order to understand practice effects in samples other than PD. The analyses of the neurocognitive outcome in single tests of the PD-DBS patients compared to the MCI group and the healthy control group revealed an outcome pattern that is very similar to the comparison of the PD-BMT group. Compared to MCI group and healthy control group PD-DBS patients showed similar gains and losses in cognition postoperatively. These results indicate that the influence of practice effects and regression to the mean effects in repeated testing designs in DBS surgery in PD patients was comparable in best treated, un-operated PD patients, clinical control groups and normal controls at similar time intervals. Thus, practice effects after DBS surgery in terms of cognitive changes are similar when using PD-BMT group, MCI group or healthy control group. These results indicate further that the effect of DBS is not beyond that one would expect, taking aging processes into consideration.

One limitation of the present study was the small sample size. Only seventy-seven participants met the inclusion criteria, which meant a small-sample number in each group. However, groups were similar for age, gender, cognitive status, verbal intelligence and depressive symptoms. Furthermore, levodopa equivalent dosage (LED), psychiatric medication (e.g antidepressants) and medication intake was not assessed before second testing. Moreover, RCI analysis does not account for the baseline differences between patients, warranting some caution when interpreting these findings. Additionally, no power analysis concerning the sample size was performed.

In summary, the present study investigated cognitive outcome in single tests one year after DBS compared to medically best treated PD patients, MCI patients and healthy controls using comprehensive neuropsychological testing. By comparing the reliable cognitive changes in individual cases over one year we obtained information regarding cognitive changes due to deep brain stimulation procedure. PD-DBS patients showed cognitive gains and losses indicating heterogeneous outcome after DBS for single patients when compared to PD-BMT patients. The cognitive outcome pattern was also comparable when using a MCI control group or a healthy control group. The reasons for the specific individual outcome are not yet clear and should be investigated in future studies.
